# Neutralizing Antibodies Against Rift Valley Fever Virus: Current Status and Advances

**DOI:** 10.3390/vaccines14060484

**Published:** 2026-05-29

**Authors:** Binjie Wu, Yuhan Sun, Yang Wang, Ye Wang, Yuyang Han, Yuan Wang, Wei Ye

**Affiliations:** 1 Student Brigade, School of Basic Medicine, Air Force Medical University: Fourth Military Medical University, Xi’an 710032, China; 2 Department of Microbiology, School of Basic Medicine, Air Force Medical University: Fourth Military Medical University, Xi’an 710032, China

**Keywords:** Rift Valley fever virus, neutralizing antibody, envelope glycoprotein Gn and Gc, viral entry, immune escape, zoonotic disease, antibody therapeutics

## Abstract

Background: Rift Valley fever virus (RVFV) is a mosquito-borne zoonotic pathogen that has caused repeated epidemics across Africa and the Arabian Peninsula, posing a severe and growing threat to public health and livestock. Infection in ruminants causes high neonatal mortality and catastrophic abortion storms; human disease ranges from self-limiting febrile illness to hemorrhagic fever, encephalitis, and permanent blindness. No licensed human vaccines or specific antiviral therapeutics are available, creating an urgent unmet medical need. Methods: We systematically reviewed the peer-reviewed literature on RVFV neutralizing antibodies (NAbs), extracting and synthesizing data on antibody sources, epitope specificity, in vitro neutralizing potency, in vivo protective efficacy, and molecular mechanisms of action. Results: A growing body of work has identified potent NAbs from immunized rodents, rabbits, alpacas, non-human primates, and convalescent patients. These NAbs predominantly target the Gn and Gc envelope glycoproteins. Their mechanisms include blocking host receptor (LRP1) binding, preventing the pH-dependent conformational rearrangement of the Gn–Gc complex, and directly inhibiting viral membrane fusion. Lead candidates, such as RVFV-268 and RVFV-140, achieve sub-nanogram neutralization and confer robust protection in rodent models against lethal challenge, aerosol exposure, and vertical transmission. Bispecific antibodies and combination strategies further enhance potency and the genetic barrier to viral escape. Conclusions: Substantial progress has illuminated the epitope landscape and neutralization mechanisms of RVFV, yielding promising clinical candidates. Translational challenges remain, including viral immune escape, antibody thermostability, and the need for rigorous preclinical evaluation. Future efforts should prioritize structure-guided engineering, rational antibody combinations, and testing in clinically predictive animal models.

## 1. Introduction

Rift Valley fever (RVF) is an acute zoonotic disease caused by Rift Valley fever virus (RVFV), which poses a severe and escalating threat to global public health and the livestock industry worldwide. RVFV is a mosquito-borne virus first identified in 1930 in the Rift Valley region of Kenya, where it was initially described as “enzootic hepatitis” due to its propensity to cause severe hepatic damage in ruminants, particularly sheep, before gradually spreading to multiple countries and regions across Africa [1,2]. Historically, RVFV has caused numerous large-scale epidemics across the African continent, resulting in massive livestock mortality and extensive human infections. In 2000, the virus emerged outside Africa for the first time, with well-documented outbreaks in Saudi Arabia and Yemen, marking a significant geographic expansion and directly threatening neighboring regions in Asia and Europe [3,4]. In July 2016, Liu et al. reported the first imported case of RVF in China, highlighting the risk of further global spread via international travel and trade [5]. In recent years, driven by global climate change, which has expanded the habitat range of mosquito vectors, and increased human population mobility, the risk of RVFV transboundary transmission and further geographic expansion has been significantly amplified. RVF is classified as a notifiable disease by the World Organisation for Animal Health (WOAH, formerly OIE) due to its substantial socioeconomic impact and pandemic potential [6].

RVFV is primarily transmitted through the bite of infected mosquito vectors, with Aedes and Culex species identified as the primary transmission vectors. Additionally, humans can acquire infection through direct contact with the blood, bodily fluids, or tissues of infected animals, as well as via inhalation of infectious RVFV aerosols in laboratory or field settings [7,8,9]. In susceptible animal hosts, RVFV infection manifests with a range of clinical signs including fever, anorexia, nasal discharge, bloody or malodorous diarrhea, and jaundice. Young livestock exhibit extremely high mortality rates following infection, while pregnant animals are highly susceptible to so-called “abortion storms”, with abortion rates in infected cattle and sheep reaching as high as 80% to 100% [10,11]. In humans, the incubation period of RVFV infection is typically 2 to 6 days, although cases with symptom onset within 24 h of exposure have been reported [12]. The majority of human RVFV infections present as an acute, self-limiting febrile illness, with patients usually achieving complete clinical recovery within two weeks and developing long-term protective immunity against re-infection [13]. However, approximately 1–2% of cases progress to severe, multi-systemic disease, manifesting as viral hemorrhagic fever, sight-threatening retinitis, meningoencephalitis, permanent blindness, or chronic neurological disorders [4,13]. Fatal cases of RVF most commonly result from severe hemorrhage, septic shock, and acute liver and renal failure [14].

Currently, there are no licensed human vaccines or specific antiviral therapeutics approved for the prevention or treatment of RVFV infection [7]. Neutralizing antibodies (NAbs), as a well-established and effective strategy for the prevention and treatment of viral infectious diseases, have demonstrated remarkable clinical potential and success in recent years for pathogens including chikungunya virus and SARS-CoV-2 [15,16]. As such, the development of NAbs targeting RVFV has garnered significant attention as a promising approach to address the unmet medical need for effective RVF countermeasures. The envelope glycoproteins Gn and Gc of RVFV, which assemble into an icosahedral lattice on the surface of mature virions, are the primary mediators of viral attachment, cellular entry, and pH-dependent membrane fusion, and thus represent the dominant targets for the induction and development of neutralizing antibodies [17,18]. Furthermore, the recent identification of low-density lipoprotein receptor-related protein 1 (Lrp1 in mice, LRP1 in humans) as a critical host entry factor for RVFV infection has provided critical new insights into the molecular mechanisms of viral tropism and novel opportunities for the rational design of NAb-based interventions [19,20].

In this review, we provide a comprehensive, systematic, and up-to-date overview of the current status of research on neutralizing antibodies against RVFV. We first summarize the virological characteristics of RVFV, with a focus on the structural and functional features of the viral envelope glycoproteins that serve as targets for NAbs. We then categorize and detail the reported RVFV NAbs based on their isolation sources, including those derived from immunized animals and convalescent human patients, with a focus on their in vitro binding affinity, neutralizing potency, in vivo protective efficacy in animal models, and mapped epitope specificities. We subsequently synthesize the established molecular mechanisms of action of these NAbs, supported by structural and functional studies. Finally, we address the key challenges that hinder the clinical translation of RVFV NAbs, and discuss future perspectives for advancing the development of NAb-based prophylactic and therapeutic strategies against RVFV.

## 2. Virological Characteristics of RVFV and Molecular Targets for Neutralizing Antibodies

**Figure 1:**
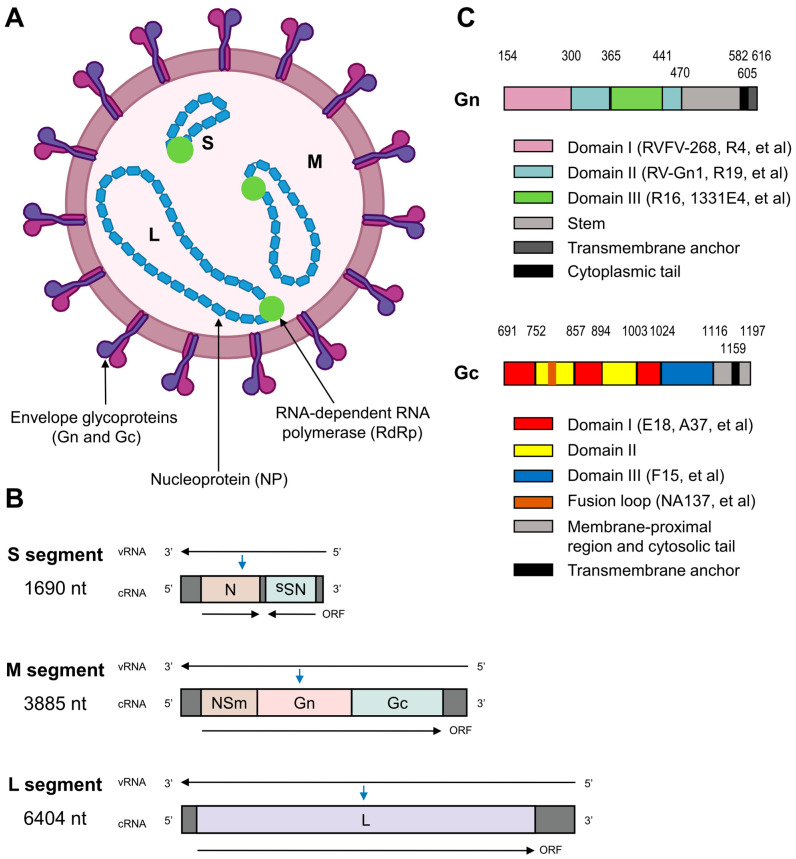
RVFV virion architecture, genome organization, and neutralizing antibody epitopes. (**A**) Schematic diagram of the RVFV virion structure. The lipid envelope (light gray) contains embedded Gn and Gc glycoprotein spikes. Inside, the ribonucleoprotein (RNP) complex and the L protein are shown. A cut-away view reveals the internal organization. (**B**) Linear representation of the three genomic segments (L, M, S). The coding regions are indicated by arrows: the L segment encodes the RNA-dependent RNA polymerase (RdRp); the M segment encodes a polyprotein precursor that is cleaved (scissors) into NSm, Gn and Gc; the S segment uses an ambisense strategy to produce the nucleoprotein (N) and the non--structural protein NSs. Segment lengths are not drawn to scale. (**C**) Domain organization of Gn and Gc with antibody binding regions. Gn is divided into a membrane-distal head (Domains A, B) and a membrane-proximal Domain C. Gc (class II fusion protein) consists of Domains I, II and III, with the fusion loop highlighted in orange. The approximate binding regions of representative neutralizing antibodies are indicated.

According to the latest classification criteria released by the International Committee on Taxonomy of Viruses (ICTV), RVFV belongs to the genus Phlebovirus within the family Phenuiviridae, with the official taxonomic name Phlebovirus riftense. It is an enveloped, single-stranded, negative-sense RNA virus (Figure 1A) [6,21]. The tripartite genome of RVFV consists of three discrete RNA segments designated L, M, and S, each encoding distinct viral proteins with specialized functions in the viral replication cycle (Figure 1B) [9].

The S segment utilizes an ambisense coding strategy to encode two proteins: the nucleoprotein (NP), which encapsidates the viral RNA genome to form the ribonucleoprotein (RNP) complex essential for viral replication and transcription, and the non-structural protein NSs [22]. NSs is well characterized as the major virulence factor of RVFV, which mediates the suppression of host innate immune responses through multiple mechanisms, including inhibition of interferon (IFN) production and disruption of host cell transcription [22]. The L segment encodes the viral RNA-dependent RNA polymerase (RdRp), the core enzyme responsible for mediating viral genome replication and mRNA transcription [23]. The M segment encodes two structural envelope glycoproteins, Gn and Gc, as well as the non-structural protein NSm, which is involved in viral assembly and modulation of host cell apoptosis [19,24].

Structural and functional studies have revealed that the Gn and Gc glycoproteins assemble into heterodimers that further oligomerize to form a highly ordered, T = 12 icosahedral lattice on the surface of the mature RVFV virion [17,18]. These two glycoproteins are the primary mediators of the early stages of the viral replication cycle, mediating viral attachment to susceptible host cells, cellular entry via endocytosis, and pH-dependent fusion between the viral envelope and the late endosomal membrane to release the viral RNP complex into the host cell cytoplasm [17,18]. The Gn glycoprotein is a type I transmembrane protein that is critical for virion maturation, assembly, and release from infected host cells [25]. Importantly, Gn contains the majority of the immunodominant antigenic epitopes that drive the induction of neutralizing antibodies in the host following infection or immunization [18,26]. The Gc glycoprotein is a class II membrane fusion protein, which is responsible for mediating the fusion between the viral envelope and the host cell late endosomal membrane, a critical step for the release of the viral genome into the cytoplasm to initiate replication [18,24,26]. Under physiological conditions in the pre-fusion state, the Gc glycoprotein is largely shielded from the extracellular environment by the membrane-distal N-terminal head domain of Gn (designated GnH), which protects the fusion-sensitive regions of Gc from premature triggering [27]. Conformational changes in the Gn-Gc complex, triggered by the acidic pH of the late endosome, are required to expose the fusion loop of Gc and initiate membrane fusion. As such, both epitopes within the Gc fusion loop and those that block the pHdependent conformational rearrangement of the Gn-Gc complex represent viable and important targets for the development of neutralizing antibodies [19]. The domain architecture of Gn and Gc is summarized in Figure 1C.

A major advance in the understanding of RVFV cellular entry was made recently by Ganaie et al., who identified LRP1 as a high-affinity, functionally critical host cell entry receptor for RVFV [19]. The study demonstrated that the Gn glycoprotein of RVFV directly binds to the extracellular domains of LRP1 to mediate viral attachment and internalization into susceptible host cells [19]. The high degree of evolutionary conservation of LRP1 across mammalian species, as well as its broad expression pattern across multiple tissues and cell types, may partially explain the wide host range and multi-organ tropism of RVFV [19]. This discovery has not only advanced our understanding of RVFV pathogenesis, but also provided a critical molecular framework for the rational design of neutralizing antibodies that block the Gn-LRP1 interaction to inhibit viral entry.

## 3. Research Progress of RVFV Neutralizing Antibodies

The earliest efforts to generate monoclonal antibodies against RVFV date back to the 1980s. In 1983, Meegan et al. used mAbs to establish Zinga virus as a strain of RVFV, providing a powerful tool for viral identification [28]. Notably, in 1986, Keegan et al. produced a panel of mAbs that exhibited neutralizing activity and were instrumental in defining early antigenic sites on the viral glycoproteins [29]. Subsequently, Besselaar and colleagues systematically characterized panels of murine mAbs against the G1 (Gn) and G2 (Gc) glycoproteins, demonstrating antigenic distinctions between wild-type and neurotropic virus strains (1991) [30], the synergistic neutralization by antibody pairs (1992) [31], and the ability of certain mAbs to neutralize at post-attachment stages of the viral entry process (1994) [32]. These early mAbs have been widely used as diagnostic and research tools within the RVFV community, and their mapped epitopes provided the first glimpse into the antigenic architecture of the virus. These pioneering studies laid the conceptual and methodological foundation for the potent neutralizing antibodies that have been developed in recent years.

Over the past decade, significant progress has been made in the discovery and characterization of RVFV neutralizing antibodies, with potent candidates isolated from immunized rodents, rabbits, alpacas, non-human primates, and convalescent human patients. More recently, in 2026, Paesen et al. determined the crystal structure of the human monoclonal antibody RVFV-379 in complex with the Gn head domain, revealing a unique k light chain binding mode that further expands our understanding of the antigenic landscape of Gn [33]. In the following sections, we integrate these historical findings with recent research advances to systematically summarize the key characteristics of these NAbs, categorized by their isolation source, including their binding affinity, in vitro neutralizing potency, in vivo protective efficacy, and mapped epitope specificities.

### 3.1. Animal-Derived Neutralizing Antibodies

Immunization of laboratory animals remains a well-established and highly effective approach for the generation of monoclonal antibodies (mAbs) against viral pathogens, including RVFV. A wide range of animal models, including mice, rabbits, alpacas, and non-human primates, have been employed for the generation of RVFV NAbs, with candidates exhibiting potent neutralizing activity and protective efficacy in preclinical models.

#### 3.1.1. Murine Monoclonal Antibodies Gn3 and Gn32

In 2020, Gutjahr et al. generated two mAbs targeting the RVFV Gn glycoprotein, designated Gn3 and Gn32, using a traditional hybridoma approach [34]. For immunization, BALB/c mice were administered three consecutive doses of 50 μg of recombinant Gn protein derived from the RVFV MP-12 vaccine strain, which comprises the 429-amino acid ectodomain of Gn fused to an N-terminal polyhistidine (His) tag, expressed and purified from *E. coli* BL21 cells, and formulated in complete Freund’s adjuvant. Following the final immunization, splenocytes were harvested from the immunized mice and fused with myeloma cells to generate hybridoma clones, from which Gn3 and Gn32 were isolated and characterized [34].

**Figure 2:**
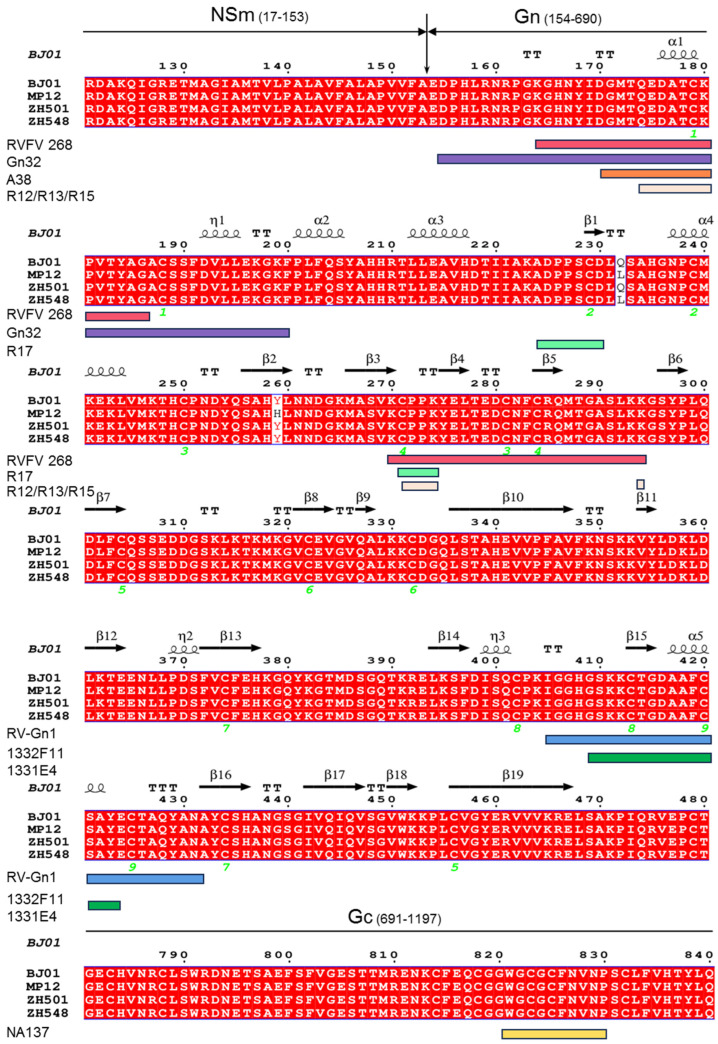
Domain organization of Gn and Gc with antibody binding regions. π-helices are indicated by large wavy lines.

ELISA-based evaluation of the binding activity of Gn3 and Gn32 to the recombinant Gn ectodomain revealed that both mAbs bind specifically to Gn, with half-maximal effective concentration (EC_50_) values of approximately 1.29 μg/mL and 0.38 μg/mL, respectively [34]. Epitope mapping experiments demonstrated that Gn32 targets the extreme N-terminal, membrane-distal region of the Gn protein, while the precise epitope recognized by Gn3 has not yet been definitively determined (Figure 2) [34]. In vitro neutralization assays using live RVFV demonstrated that only Gn3 exhibited significant neutralizing activity, with a half-maximal inhibitory concentration (IC_50_) value of 33.0 μg/mL, while Gn32 lacked intrinsic neutralizing activity. Notably, when Gn3 and Gn32 were co-incubated with the virus, the neutralizing potency was enhanced, with the IC_50_ value reduced to 24.6 μg/mL, indicating a potential synergistic effect between the two mAbs [34].

Multiple sequence alignment of the Gn Gc region from four RVFV strains (ZH501, MP12, ZH548, BJ01). The numbering corresponds to the full—length M segment polyprotein of ZH501. Conserved residues are shaded in gray. Secondary structure elements predicted from the Gn crystal structure (PDB: 5Y0W) are displayed above the alignment as follows:

- α—helices are indicated by the coiled symbol above the alignment.
- 3_10_—helices are indicated by η (eta).
- β—strands are indicated by β (with sequential numbering, e.g., β 1, β 2…).
- β—turns are indicated by TT.
- π-urns ar are indicated by large wavy lines.

Green numbers below the alignment indicate conserved cysteine (Cys) residues involved in disulfide bond formation.

The boundary between Gn (154–690) and Gc (691–1128) is marked by horizontal bars. The approximate binding regions of representative neutralizing antibodies are color—coded below the alignment: RVFV—268 (red), Gn32 (purple), R17 (green), R12/R13/R15 (light orange), RV—Gn1 (blue), A38 (orange), and NA137 (yellow; binds to the Gc fusion loop, which lies outside the aligned Gn region and is indicated schematically by a yellow bar below the Gc annotation). Antibodies that recognize conformational or quaternary epitopes (e.g., RVFV—140) are discussed in the text.

The in vivo protective efficacy of Gn3 and Gn32, alone and in combination, was evaluated in a lethal BALB/c mouse challenge model using the virulent RVFV 35/74 strain. Mice were administered 200 μg of each mAb via tail vein injection either 30 min before or 30 min after viral challenge. Treatment with Gn32 alone, either pre- or post-exposure, did not confer significant protection against lethal challenge, with survival rates of 8% in the pre-exposure group and 25% in the post-exposure group. Monotherapy with the neutralizing antibody Gn3 provided moderate protection, with an overall survival rate of 58.3% across both pre- and post-exposure settings. Strikingly, the combination of Gn3 and Gn32 provided nearly complete protection against lethal challenge, with a survival rate of 83.3% in the pre-exposure treatment group and 100% survival in the post-exposure treatment group, compared to a survival rate of only 16.67% in the untreated control group [34]. Postmortem analysis of the liver and brain tissues, as well as coagulation parameter analysis, revealed that mice treated with the combination of Gn3 and Gn32 exhibited significantly reduced viral RNA loads in both organs and peripheral blood compared to the untreated control group, consistent with the observed survival benefit [34].

These results suggest that the synergistic neutralization and protective effect of Gn3 and Gn32 is mediated by Gn32 binding to the N-terminal region of Gn, which induces a conformational change in the glycoprotein that increases the accessibility of the epitope recognized by Gn3, thereby enhancing its neutralizing activity [34]. This finding provides important preclinical proof-of-concept for the use of antibody combinations targeting distinct epitopes on the Gn glycoprotein to enhance the protective efficacy of NAb-based interventions against RVFV (Table 1).

#### 3.1.2. Murine Monoclonal Antibodies Gc1-Gc9 and Gn1-Gn14

More recently, Ronit Rosenfeld et al. employed high-throughput single-cell transcriptomic and B cell receptor (BCR) sequencing technology to isolate and characterize a panel of 23 recombinant mAbs targeting the RVFV glycoproteins, designated Gc1-Gc9 (targeting Gc) and Gn1-Gn14 (targeting Gn) [41]. For immunization, female BALB/c mice (6–8 weeks of age) were primed via subcutaneous injection with 1 × 10^6^ plaque-forming units (PFU) of recombinant RVFV strain rMP-12-GFP (a recombinant MP-12 strain expressing green fluorescent protein, GFP), followed by two booster immunizations at monthly intervals with 4 × 10^7^ PFU and 1 × 10^9^ PFU of rMP-12-GFP, respectively. Subsequently, the mice received three additional subcutaneous immunizations at 4-week intervals with 150 μg of recombinant RVFV glycoprotein (rGc or rGn) formulated in incomplete Freund’s adjuvant (IFA). Serum samples were collected prior to the initial immunization and approximately two weeks after each immunization to monitor the development of antigen-specific antibody responses [41].

Following the final immunization, CD19^+^/IgG^+^/antigen^+^ memory B cells were isolated from the splenocytes of the immunized mice via fluorescence-activated cell sorting (FACS). Single-—cell transcriptome sequencing and paired BCR sequencing were performed on the sorted memory B cells using the 10× Genomics platform. Through bioinformatic analysis, BCR pairs with high clonal abundance and antigen specificity were selected for cloning and recombinant expression as full-length IgG mAbs [41].

The antigen-binding activity and affinity of the resulting recombinant mAbs were evaluated via ELISA and biolayer interferometry (BLI). Approximately half of the isolated mAbs exhibited specific, high-affinity binding to their target antigens. For the Gc-targeting mAbs, the equilibrium dissociation constant (Kd) values ranged from 2.1 nM to 32 nM, with Gc3, Gc9, and Gc4 exhibiting the highest binding affinities, and mAb Gc1 showing the lowest affinity. For the Gn-targeting mAbs, Kd values ranged from 0.65 nM to 8 nM, with Gn14 and Gn4 demonstrating sub-nanomolar binding affinities, and Gn8 exhibiting the lowest affinity [41]. This study demonstrated the utility and efficiency of high-throughput single-cell sequencing technology for the rapid discovery of antigen-specific mAbs, providing a powerful platform for the accelerated development of antibody countermeasures against emerging viral threats such as RVFV (Table 1) [41].

#### 3.1.3. Rabbit-Derived Monoclonal Antibodies RV-Gn1, RV-Gn2, and RV-Gn3

In 2018, Allen et al. isolated three potent RVFV-neutralizing mAbs, designated RV-Gn1, RV-Gn2, and RV-Gn3, from immunized New Zealand white rabbits [35]. For immunization, male New Zealand white rabbits (8–10 weeks of age) were administered four doses of 120 μg of full-length recombinant RVFV Gn ectodomain protein mixed with Adjuplex adjuvant at a 5:1 protein:adjuvant ratio, at weeks 0, 4, 8, and 16. Seven days after the final immunization, splenocytes were harvested for the generation of rabbit hybridomas, and peripheral blood mononuclear cells (PBMCs) were simultaneously isolated for single B cell sorting [35].

Two mAbs with high-affinity binding to RVFV Gn, RV-Gn1 and RV-Gn2, were isolated from the rabbit hybridoma clones, while a third high-affinity Gn-binding mAb, RV-Gn3, was isolated from sorted CD3^−^ IgM^−^ IgG^+^ RVFV Gn^+^ B cells from the PBMCs of the immunized rabbits, via reverse transcription and PCR amplification of the antibody light and heavy chain variable region genes [35]. Plaque reduction neutralization tests (PRNT) performed in Vero cells demonstrated that all three mAbs efficiently neutralized live RVFV, with IC_50_ values ranging from 2.1 to 3.0 μg/mL [35]. Subsequent sequence analysis revealed that the complementarity-determining region (CDR) sequences within the antigen-binding fragment (Fab) regions of the three mAbs are highly conserved, and analysis of their variable (V), joining (J), and diversity (D) gene segments indicated that the three mAbs are derived from germline-related clonal lineages. ELISA competition assays further confirmed that the three mAbs target an overlapping, common epitope on the Gn glycoprotein, thus classifying them as a single family of RVFV-neutralizing antibodies [35].

The in vivo protective efficacy of the lead candidate, RV-Gn1, was evaluated in a lethal BALB/c mouse challenge model using the highly virulent RVFV ZH501 strain. Intravenous injection of either 10 μg or 200 μg of RV-Gn1 at 8 h prior to challenge with 20 PFU of RVFV ZH501 fully protected the mice from lethal infection, with 100% survival in the antibody-treated groups, while all mice in the untreated RVFV control group succumbed to infection. Notably, a 40% survival rate was observed in the IgG isotype control group in this study [35].

Previous structural studies of the RVFV Gn ectodomain had revealed that the Gn protein is organized into three distinct domains arranged in a triangular configuration: domain A (residues 154–300), domain B (residues 366–439), and a β-ribbon domain (residues 301–365 and 440–469) [25]. To elucidate the molecular basis of neutralization by RV-Gn1, the investigators solved the co-crystal structure of the RV-Gn1 Fab in complex with the RVFV Gn ectodomain [35]. The structure revealed that RV-Gn1 binds primarily to the membrane-distal domain B of RVFV Gn, with the binding interface concentrated on two flexible loop regions spanning residues 405–431 and 423–431 within domain B (Figure 2). Structural analysis revealed that binding of RV-Gn1 to this region sterically blocks the exposure of the fusion loop located within the Gc glycoprotein, thereby preventing the pH-dependent membrane fusion event required for viral entry into target cells. Detailed analysis of the binding interface demonstrated that the heavy chain CDRs of RV-Gn1 contribute 12 of the 15 resolved hydrogen bonds between the antibody and Gn, while the light chain CDRs contribute only 3, indicating that the heavy chain is the primary mediator of antigen recognition. Notably, this antigen–antibody binding mode is highly similar to that previously observed for the neutralizing antibody mAb4-5 targeting the Gn glycoprotein of severe fever with thrombocytopenia syndrome virus (SFTSV), another member of the Phlebovirus genus, despite only 24% sequence conservation between the Gn proteins of these two distantly related phleboviruses. This striking observation suggests that the host antibody response targeting the membrane-distal domain B of Gn may represent a conserved, convergent feature of the humoral immune response against phlebovirus infection (Table 1) [35].

#### 3.1.4. Alpaca-Derived Nanobody NA137 and Bispecific Antibody E2-NA137

In 2025, Zhao et al. reported the identification and characterization of a potent Gc-targeting nanobody (NA137) and a bispecific antibody derivative (E2-NA137) with enhanced neutralizing potency against RVFV [42]. For immunization, adult alpacas were administered four intramuscular doses of 2 mL mRNA encoding the Gc_691—1119_ ectodomain (500 μg per dose) at 14-day intervals. Serum anti-Gc-specific antibody titers were monitored by ELISA throughout the immunization regimen, and a phage display nanobody library was constructed from the peripheral B cells of the immunized alpacas [42].

After four rounds of phage display biopanning against the recombinant Gc protein, nanobody NA137 was identified from 92 positive clones, which exhibited robust neutralizing activity against RVFV with an IC_50_ value of 2.701 μg/mL [42]. ELISA and surface plasmon resonance (SPR) validation confirmed that NA137 binds specifically to the Gc protein with nanomolar affinity, with a Kd value of 1.21 nM. To further improve potency, they constructed a bispecific antibody, E2-NA137, by fusing the VHH domain of NA137 to the heavy chain of E2, a Gn targeting neutralizing IgG, in an IgG VHH format. This bispecific design simultaneously targets both Gn and Gc glycoproteins of RVFV, resulting in a 270-fold enhancement in neutralizing potency compared to the parental NA137 nanobody, with an IC_50_ value of 0.010 μg/mL, and an ultra-high binding affinity with a Kd value of 0.00489 nM [42].

The in vivo protective efficacy of NA137 and E2-NA137 was evaluated in an interferon-α/β receptor-deficient (A129) mouse model, which is highly susceptible to RVFV infection. In the pre-exposure prophylaxis group, intraperitoneal administration of 200 μg of either NA137 or E2-NA137 at 24 h prior to viral challenge resulted in an 80% survival rate. In the post-exposure treatment group, where antibodies were administered 24 h after infection, the E2-NA137-treated group achieved a 60% survival rate, which was significantly superior to the 20% survival rate observed in the NA137 monotherapy group [42]. Histopathological analysis of the brain, liver, and spleen tissues revealed that antibody-treated mice did not develop the typical necrotic lesions observed in the PBS-treated control group, and immunohistochemical staining detected no viral antigen in the organs of surviving mice. Quantitative real-time PCR (qRT-PCR) confirmed that antibody treatment significantly reduced viral loads in the liver (*p* < 0.0001), with the E2-NA137-treated group exhibiting a 3-log reduction in viral RNA copies in the spleen compared to the control group. These results collectively demonstrate that both NA137 and E2-NA137 provide robust protection against lethal RVFV challenge in vivo, with the bispecific E2-NA137 exhibiting superior therapeutic efficacy [42].

To elucidate the structural basis of antigen recognition, the investigators used AlphaFold 3 to predict the three-dimensional structure of the NA137-Gc and E2-Gn antigen–antibody complexes, with in-depth structural analysis performed using ChimeraX 1.8 molecular visualization software [42]. This analysis revealed that the key epitope residues for NA137 binding to Gc include R85, R86, C87, W131, F136, and D271, which are located within the functionally critical fusion loop and adjacent regions of the Gc protein. For the E2-Gn complex, the key epitope residues involved in antibody binding include G18, D77, and L79 on the Gn glycoprotein. These results supports a model in which E2 binding to Gn induces a conformational change in the Gn-Gc complex that cooperatively exposes the Gc epitope targeted by NA137, resulting in synergistic neutralization. This dual-targeting mechanism not only enhances neutralizing potency, but also significantly reduces the risk of viral immune escape, as the virus would need to acquire simultaneous mutations in two distinct epitopes to evade neutralization. Further structural analysis via cryo-electron microscopy (cryo-EM) is warranted to fully validate this proposed mechanism (Table 1) [42].

#### 3.1.5. Non-Human Primate-Derived Monoclonal Antibodies 1331E4 and 1332F11

In 2020, Meng et al. isolated two potent RVFV-neutralizing mAbs, 1331E4 and 1332F11, from rhesus macaques immunized with a recombinant human adenovirus type 4 vaccine expressing codon-optimized RVFV Gn and Gc proteins (rHAdV4-GnGcopt) [40]. PBMCs were isolated from the immunized macaques, and Gn protein-specific memory B cells were sorted via FACS. Antibody variable region genes from 8 κ subtype and 7 λ subtype clones were cloned and expressed as full-length human IgG1 mAbs, among which two antibodies, 1332F11 (κ subtype) and 1331E4 (λ subtype), exhibited robust neutralizing activity against live RVFV in vitro [40].

In vitro virus neutralization assays demonstrated that 1332F11 and 1331E4 have IC_50_ values of 167.8 ng/mL and 338.8 ng/mL, respectively [40]. BLI analysis revealed that 1331E4 binds to the RVFV Gn protein with a Kd value of 0.938 nM, which is 17-fold lower than that of 1332F11 (16 nM), indicating that 1331E4 has a significantly higher binding affinity for the Gn glycoprotein. Western blot, ELISA, and in silico antigen–antibody molecular docking models demonstrated that both antibodies recognize the Gn9a peptide spanning amino acid residues 409–423, which is located within domain III of the Gn glycoprotein. Although the two antibodies bind to this region in different orientations, their key binding residues are highly similar. The docking models further revealed that the antibody binding sites are located in close proximity to the fusion loop of the Gc glycoprotein, suggesting that these antibodies may neutralize RVFV by preventing the pH-dependent conformational rearrangement of the Gn-Gc complex required for membrane fusion. However, further in vivo studies are needed to validate the protective efficacy of these two NAbs in animal models of RVFV infection (Table 1) [40].

#### 3.1.6. Non-Human Primate-Derived Monoclonal Antibodies A13 and A38

In 2026, Hao et al. reported the isolation and comprehensive characterization of a panel of potent RVFV-neutralizing mAbs from rhesus macaques immunized with Ad4-GnGc or Ad5-GnGc adenovirus vector vaccines [38]. PBMCs and serum samples were collected from the immunized macaques, and single memory B cell sorting technology was used to isolate 77 Gn-targeting mAbs, of which 20 exhibited robust neutralizing activity against RVFV in vitro [38].

Competition binding assays and BLI analysis revealed that these neutralizing antibodies can be categorized into two major groups: Group A (10 antibodies) targeting subdomain I of the Gn protein, and Group B (10 antibodies) targeting subdomain III of the Gn protein. Group B was further subdivided into two subgroups, B1 and B2, with B1 antibodies able to interfere with the binding of B2 antibodies to Gn, while the reverse was not observed [38]. SPR analysis demonstrated that the majority of these neutralizing antibodies exhibit high binding affinity for the Gn protein, with Kd values ranging from 0.37 to 211.67 nM. Microneutralization assays revealed that 12 of the isolated neutralizing antibodies have IC_50_ values below 10 ng/mL against the RVFV rMP-12 strain. Among these, the Group A representative antibody A38 exhibited the strongest neutralizing activity, with an IC_50_ value of 0.76 ng/mL, while the Group B representative antibody A13 exhibited the highest binding affinity, with a Kd value of 0.40 nM. Alanine scanning mutagenesis identified 11 key amino acid residues (including D170 and T173) required for A38 binding to Gn subdomain I, and 6 key amino acid residues (including G409 and C413) required for A13 binding to Gn subdomain III (Figure 2) [38].

The in vivo protective efficacy of A38 and A13 was evaluated in the A129 mouse model of lethal RVFV infection. Mice were administered 200 μg (high dose) or 20 μg (low dose) of A38 or A13 via intraperitoneal injection either 24 h before or 24 h after challenge with 2 × 10^4^ TCID_50_ of the RVFV rMP-12 strain [38]. For prophylactic administration, high-dose A38 and A13 both achieved 100% survival of the challenged mice. Low-dose A38 maintained 100% survival, while low-dose A13 resulted in only 1 out of 8 mice surviving. For therapeutic administration, high-dose A13 and both high- and low-dose A38 achieved 100% survival, while the low-dose A13 group showed 6 out of 8 mice surviving, with a significantly prolonged time to death for the animals that succumbed to infection. All antibody-treated groups exhibited significantly reduced liver viral loads compared to the PBS control group, and surviving mice showed only mild, transient body weight fluctuations followed by rapid recovery [38].

Mechanistic studies revealed that A38 exerts its neutralizing effects through a dual mechanism of action: it both blocks the attachment of RVFV to host cells and inhibits viral membrane fusion [38]. In contrast, A13 achieves neutralization primarily through the inhibition of membrane fusion. Molecular docking analysis indicated that the Fab fragment of A38 binds to both subdomains I and III of the Gn protein, stabilizing the pre-fusion conformation of the Gn-Gc complex through hydrogen bonds and hydrophobic interactions, thereby preventing the exposure of the Gc fusion loop. The Fab fragment of A13 recognizes a conformational epitope spanning all three subdomains of Gn, preventing the conformational rearrangement of the Gn-Gc complex through steric hindrance, thereby inhibiting membrane fusion. This study represents the first identification of RVFV-neutralizing antibodies capable of simultaneously blocking multiple stages of the viral entry process, providing high-quality candidate molecules for the development of therapeutic antibodies against RVFV (Table 1) [38].

### 3.2. Convalescent Patient-Derived Neutralizing Antibodies

Fully human neutralizing antibodies isolated from the memory B cells of convalescent RVF patients represent the most promising candidates for clinical translation, as they exhibit low immunogenicity in humans and are naturally selected for high specificity and potency against the native virus. In this section, we summarize the key characteristics of fully human RVFV NAbs isolated from convalescent patients, with a focus on their neutralizing potency, structural mechanisms of action, and in vivo protective efficacy.

#### 3.2.1. Human Monoclonal Antibodies R4, R12, R13, R15, R16, R17, R19, R22, and R5

In 2019, Wang et al. isolated a panel of nine fully human RVFV-specific mAbs from the first imported RVF case in China, a patient who had returned to China from Angola and fully recovered from infection [39]. PBMCs were isolated from the convalescent patient, incubated with 100 nM His-tagged recombinant Gn and Gc proteins, and antigen-specific memory B cells were sorted via flow cytometry. The antibody variable region genes were amplified via reverse transcription PCR, cloned, and expressed as full-—length human IgG1 mAbs, yielding nine specific antibodies designated R4, R12, R13, R15, R16, R17, R19, R22 (all targeting Gn), and R5 (targeting Gc) [39].

PRNT assays performed in Vero cells revealed that the Gn-targeting mAbs exhibited significantly stronger neutralizing activity against RVFV compared to the Gc-targeting mAb R5 [39]. Among the Gn-targeting mAbs, R15 and R16 exhibited the highest neutralizing potency, with IC_50_ values of 0.53 ± 0.25 ng/mL and 0.29 ± 0.09 ng/mL, respectively. R12 and R17 also exhibited potent neutralizing activity, with IC_50_ values of 1.85 ng/mL and 2.53 ng/mL, respectively. R4, R13, R19, and R22 showed moderate neutralizing activity, with IC_50_ values ranging from 46.8 to 73.7 ng/mL. The Gc-targeting mAb R5 exhibited the weakest neutralizing activity, with an IC_50_ value of 1370 ± 530 ng/mL [39].

The in vivo protective efficacy of these nine mAbs was evaluated in a lethal BALB/c mouse challenge model [39]. In the post-exposure treatment group, mice were administered 10 mg/kg of each mAb via intraperitoneal injection 24 h after challenge with 1 × 10^3^ PFU of RVFV. After 14 days of observation, all mice in the eight Gn-targeting mAb treatment groups survived, with the exception of one mouse in the R22 group that died on day 11 post-infection. In contrast, only 1 out of 5 mice in the R5 treatment group survived, while all mice in the untreated control group succumbed to infection within 5 days post-challenge. In the pre-exposure prophylaxis group, mice were administered 10 mg/kg of each mAb 24 h prior to viral challenge. After 14 days of observation, all mice in the Gn-targeting mAb treatment groups survived, while 4 out of 5 mice in the R5-treated group died, and all control mice succumbed to infection within 5 days. Subsequent analysis revealed that despite its relatively low neutralizing potency, R5 was able to prevent RVFV infection at high concentrations and contributed to viral clearance in vivo, suggesting that the inclusion of both Gn and Gc antigens in vaccine design may elicit broader protective immunity compared to Gn alone [39].

To elucidate the molecular basis of neutralization, the investigators solved the co-crystal structures of RVFV Gn in complex with the Fab fragments of R12, R13, and R15, as well as the single-chain variable fragment (scFv) of R17, using X-ray crystallography, with additional validation via SPR [39]. Co-crystal structures and mutational analysis revealed that the epitopes of R12, R13, and R15 are substantially overlapping, primarily comprising two key antigenic regions: epitope A (Gn-ΔA), consisting of the η2α1 helix (173-TQEDATCK-180) and K294, and epitope B (Gn-ΔB), consisting of the β3/β4 loop (271-CPPK274). R12 primarily forms an electrostatic interaction patch with N176 and neighboring residues including K164, H166, Y168, K180, and E174 of Gn. R13 engages in hydrophilic interactions with the α1 helix (176-DAT-178), K180, and K294. R15 targets residues Q174, D176, K180, and K294 within epitope A. R17 primarily binds to epitope B and the β1 chain (223-KADPPSCD-230), interacting with residues K223, D225, D230, and S228 of Gn (Figure 2). With the exception of CDR1 in the heavy chain of R13, all CDRs of the heavy and light chains of R12, R13, R15, and R17 directly participate in interactions with RVFV Gn, neutralizing the virus by blocking the interaction between the Gn glycoprotein and host cell receptors, thereby preventing viral entry into target cells [39].

Further mutational analysis of the mAbs without resolved co-crystal structures revealed that R4 maintains interaction with Gn-ΔA but cannot bind Gn-ΔB; R16 cannot bind either Gn-ΔA or Gn-ΔB but interacts normally with Gn mutants in the C region; R19 binds Gn-ΔB but cannot interact with Gn-ΔA; and R22 can bind both Gn-ΔA and Gn-ΔB, with specific mutations attenuating its binding, requiring further analysis of its binding characteristics. Western blot analysis indicated that none of the Gn mAbs or the Gc mAb R5 recognize linear epitopes, indicating that they all target conformational epitopes on the native glycoprotein (Table 1) [39].

#### 3.2.2. Human Monoclonal Antibody RVFV-268

In 2021, Chapman et al. isolated a panel of 20 fully human neutralizing mAbs from B cells obtained from individuals in Kenya who had either naturally recovered from RVFV infection or been immunized with the live-attenuated MP-12 vaccine [17]. B cells were transformed in vitro with Epstein–Barr virus (EBV) to generate B cell-derived lymphoblastoid cell lines (LCLs), which were screened for RVFV-neutralizing activity via focus-forming assay (FFA). Positive LCLs were electrofused with a non-secretory myeloma cell line, and the resulting hybridoma cells were cloned via flow cytometry, yielding 20 hybridoma clones secreting fully human mAbs with potent RVFV-neutralizing activity [17]. Among these mAbs, several lead candidates, including RVFV-268, RVFV-140, and RVFV-226, exhibited robust neutralizing activity in vitro and potent protective efficacy in mouse models, highlighting their potential as antiviral therapeutics for RVFV.

Through competitive binding analysis and mutant screening, the investigators identified four major antigenic sites on the RVFV Gn glycoprotein, with many of the most potent neutralizing antibodies binding to site A (domain A) of Gn [17]. Among these, the lead candidate RVFV-268, which also binds to Gn domain A, exhibited exceptional neutralizing potency, with an IC_50_ value of 0.2 ng/mL in in vitro neutralization assays, making it one of the most potent RVFV-neutralizing antibodies reported to date [36].

The in vivo protective efficacy of RVFV-268 was extensively evaluated in multiple animal models [17]. In a prophylactic setting, intraperitoneal administration of either 10 μg or 200 μg of RVFV-268 at 2 h prior to challenge with 300 PFU of the virulent RVFV ZH501 strain fully protected inbred C57BL/6 mice from lethal infection. In a post-exposure therapeutic setting, BALB/c mice were administered 200 μg of RVFV-268 via intraperitoneal injection either 2 or 4 days after RVFV infection, and were euthanized on day 5 for assessment of viral titers in the serum, liver, and spleen. The results demonstrated that administration of RVFV-268 at 2 days post-infection resulted in a significant therapeutic benefit, with a marked reduction in viral loads in all tissues, while administration at 4 days post-infection resulted in a significantly reduced survival benefit [17].

Structural analysis via X-ray crystallography and cryo-EM revealed that RVFV-268 primarily recognizes the membrane-distal GnH region of the RVFV Gn glycoprotein [17]. The two epitopes recognized by the bivalent IgG are proximal to each other and exposed on the surface of the pre-fusion Gn-Gc complex, allowing recognition without altering the ultrastructure of the Gn-Gc complex, and enabling both Fab regions of a single RVFV-268 IgG molecule to simultaneously bind to adjacent Gn proteins on the viral surface. BLI analysis revealed that RVFV-268 does not directly compete with the binding of Gn to the LRP1 host receptor. Instead, through its bivalent binding mode, it cross-links Gn proteins on the viral surface, thereby masking the LRP1 binding interface and blocking the interaction between the virus and host cell receptors. Consistent with this mechanism, when RVFV-268 was converted from a bivalent IgG format to a monovalent Fab format, its neutralizing activity was completely abolished [19].

In 2024, Connors et al. further evaluated the protective efficacy of RVFV-268 in a Lewis rat model of aerosolized RVFV infection, which recapitulates the severe neurological disease observed in a subset of human RVF cases [7]. Administration of 10 mg/kg of RVFV-268 at 24 h prior to aerosol exposure resulted in a survival rate of up to 72%, with no neurological symptoms observed in any of the surviving rats. Viral RNA loads and infectious viral titers in the brain and liver tissues of the treated rats were significantly reduced, with no infectious virus detected even at 5 days post-infection [7]. Furthermore, RVFV-268 exhibited potent efficacy in preventing vertical transmission of RVFV in a rat model of congenital infection. Following intraperitoneal injection, RVFV-268 rapidly crossed the placenta into fetal tissues, where it persisted for at least 24 h, protecting both the maternal host and fetuses from infection. Notably, even when administered within 24 h after maternal RVFV infection, RVFV-268 was able to prevent vertical transmission, demonstrating its potential for both prophylactic and therapeutic applications in the prevention of congenital RVF [36].

However, despite its excellent neutralizing potency of RVFV-268, several limitations still need to be considered before clinical application. In vitro neutralization assays in HEK-293 and HepG2 cell lines have shown that RVFV-268 can leave a small fraction of virus unneutralized [19]. However, this cell-type-specific observation does not appear to compromise in vivo efficacy: RVFV-268 confers robust protection across multiple animal models, with efficacy comparable to or exceeding that of other clinical candidates. In addition, in vitro experiments assessing the susceptibility of RVFV-268 to natural viral mutations indicated that the antibody retains only partial binding capacity to Gn proteins with T173L or E175G mutations, and completely loses binding ability to Gn proteins harboring the K294E-D230N double mutation [19]. Further in vitro competitive binding experiments confirmed that the activities of antibodies targeting the Gn domain A (including RVFV-268) are highly dependent on two amino acid pockets on Gn protein (residues 164–186 and 270–294), which makes them highly susceptible to immune escape by this highly mutable RNA virus (Table 1) [19]. Nevertheless, this in vitro phenomenon does not seem to result in reduced in vivo efficacy. RVFV-268 exerts potent protective effects in a variety of animal models, with efficacy equivalent to or even better than that of other major candidate antibodies. This discrepancy may be attributed to cell type-specific entry pathways that are bypassed in vivo, or the contribution of Fc-mediated effector functions.

#### 3.2.3. Human Monoclonal Antibody RVFV-140

In addition to RVFV-268, RVFV-140 is another key fully human neutralizing mAb discovered by Chapman et al. from the same cohort of convalescent and vaccinated individuals [19]. FFA-based in vitro neutralization assays using the RVFV MP-12 strain demonstrated that RVFV-140 has an IC_50_ value of approximately 13 ng/mL, which is lower than that of RVFV-268, but it exhibits distinct and advantageous functional characteristics [19].

Previous studies had suggested that antibodies targeting Gn domain A, such as RVFV-268, mediate neutralization primarily by blocking viral binding to receptors such as DC-SIGN. However, cells and tissues lacking DC-SIGN expression remain susceptible to RVFV infection, leading the investigators to hypothesize and subsequently validate the existence of an alternative neutralization mechanism: inhibition of viral membrane fusion [17,19]. A critical step in RVFV entry into host cells is the fusion of the viral envelope with the late endosomal membrane, which is triggered by the acidic pH of the endosome, inducing a conformational rearrangement of the Gn-Gc complex that exposes the Gc fusion loop to mediate membrane fusion. Studies revealed that mAbs targeting the Gn-Gc complex and some Gn domain A-specific mAbs can inhibit RVFV membrane fusion, thereby preventing viral infection. Notably, mAbs targeting quaternary epitopes on the assembled Gn-Gc complex exhibited particularly potent fusion-inhibiting activity, with IC_50_ values below 10 ng/mL [19]. This fusion-inhibiting neutralization mechanism is thought to be mediated by antibodies binding to the pre-fusion Gn-Gc complex and obstructing the conformational rearrangement required for membrane fusion, thereby preventing the exposure of the Gc fusion loop following endosomal acidification [19].

Fusion-from-without (FFWO) assays, which measure cell–cell fusion independent of viral replication, demonstrated that RVFV-140 neutralizes RVFV by potently inhibiting membrane fusion, specifically by preventing the pH-dependent conformational rearrangement of the viral Gn-Gc glycoproteins required for the fusion process [19]. Although the IC_50_ value of RVFV-140 is higher than that of RVFV-268, RVFV-140 achieved 100% neutralization of viral infection across all tested cell lines, including HEK-293 (kidney), SH-SY5Y (neuroblastoma), and HepG2 (liver) cells, whereas RVFV-268 left a residual fraction of unneutralized virus in these cell lines [19]. This indicates that RVFV-140 may provide more comprehensive protection across a broader range of host tissues compared to RVFV-268. Furthermore, in the Lewis rat aerosol challenge model, administration of 10 mg/kg of RVFV-140 at 24 h prior to exposure achieved a survival rate of 83%, the highest among all tested mAbs [7]. Additionally, compared to RVFV-268, the reduction in neutralizing potency of the monovalent Fab format of RVFV-140 was significantly less pronounced, further confirming the distinct neutralization mechanisms of these two antibodies [19].

In 2025, Zhang et al. resolved the near-atomic resolution cryo-EM structure of the RVFV GnGc hexamer in complex with the Fab fragment of RVFV-140 (Fab140), providing a definitive structural elucidation of the membrane fusion inhibition mechanism of this antibody [37]. This study confirmed that RVFV-140 does not bind to monomeric Gn or Gc proteins, but instead specifically recognizes a quaternary conformational epitope on the assembled GnGc hexameric complex. Fab140 binds to the top crown region of the GnGc hexamer, cross-linking two adjacent Gn head domains through 13 hydrogen bonds and 4 salt bridges. The light chain of Fab140 interacts exclusively with domain A of one Gn monomer, while the heavy chain simultaneously binds domain A of the same Gn monomer and domain B of the adjacent Gn monomer. The conformational epitope recognized by RVFV-140 covers a buried surface area of approximately 800 Å^2^, and shows no spatial overlap with the LRP1 receptor binding region on Gn, confirming that its neutralizing activity is not mediated by blocking virus-receptor binding [37].

During RVFV invasion, the acidic environment of the late endosome triggers protonation of the pH-sensitive residue H540 in the C domain of Gn, which generates electrostatic repulsion with surrounding positively charged residues, promoting the release of the Gn C domain and initiating the conformational rearrangement of the GnGc complex [37]. This rearrangement provides the structural space required for the exposure of the Gc fusion loop, the extension of the Gc ectodomain, and the subsequent virus–host membrane fusion. By cross-linking adjacent Gn molecules in the pre-fusion hexamer, RVFV-140 creates a steric block that directly prevents the pH-dependent conformational rearrangement of Gn, thereby blocking the exposure of the Gc fusion loop and the subsequent membrane fusion process. Additionally, antibody binding may induce deformation of the viral particle, further enhancing its neutralizing efficacy. Most importantly, RVFV-140 binding stabilizes the pre-fusion assembly of the GnGc hexamer, fundamentally blocking the conformational transition of the viral glycoproteins from the pre-fusion to the post-fusion state. This structural stabilization represents the core mechanistic basis for the potent and broad membrane fusion-inhibiting activity of RVFV-140, and provides a structural blueprint for the rational design of next-generation fusion-inhibiting antibodies against RVFV and other phleboviruses [37]. Collectively, the characterization of RVFV-140 has revealed that targeting quaternary conformational epitopes on the assembled GnGc complex represents a highly effective strategy for the development of broadly neutralizing antibodies against RVFV, with a higher genetic barrier to viral escape compared to antibodies targeting linear or monomeric epitopes (Table 1).

## 4. Challenges and Future Perspectives

Despite the remarkable progress in the discovery and preclinical development of RVFV-neutralizing antibodies over the past decade, several critical challenges remain that must be addressed to facilitate the clinical translation of these promising candidates into licensed prophylactic and therapeutic interventions for human RVF. In this section, we systematically discuss these key challenges, and highlight future research directions to overcome these barriers and advance the field.

### 4.1. Viral Immune Escape and Epitope Conservation

One of the most significant and well-recognized challenges for the development of antibody-based therapeutics against RNA viruses is the risk of viral immune escape driven by the high mutation rate of the viral RNA-dependent RNA polymerase, which lacks proofreading activity. This challenge is particularly acute for RVFV, as evidenced by the well-characterized escape mutations of the lead clinical candidate RVFV-268. Despite its exceptional in vitro neutralizing potency (IC_50_ = 0.2 ng/mL), RVFV-268 exhibits reduced binding to Gn proteins carrying the naturally occurring T173L or E175G mutations, and completely loses binding and neutralizing activity against the K294E-D230N double mutant [19]. Structural analysis has confirmed that antibodies targeting the immunodominant domain A of Gn, including RVFV-268, R12, R13, and R15, are critically dependent on two discrete amino acid pockets within Gn (residues 164–186 and 270–294) (Figure 2) [19,33,39]. These regions are highly exposed on the virion surface and are the primary targets of the host humoral immune response, but they are also genetically plastic, with minimal functional constraints on mutation, making them particularly vulnerable to immune escape.

This highlights a critical trade-off in epitope selection for therapeutic antibody development: antibodies targeting highly accessible, immunodominant, but genetically variable regions (such as Gn domain A) can achieve exceptional neutralizing potency, but this often comes at the expense of reduced breadth and increased susceptibility to viral escape mutants. In contrast, antibodies targeting functionally constrained, highly conserved regions of the viral glycoproteins—such as the Gc fusion loop, or quaternary conformational epitopes that are dependent on the intact, assembled Gn-Gc complex—may offer a higher genetic barrier to viral escape, as mutations in these regions would likely impair viral fitness and infectivity. For example, RVFV-140 recognizes a quaternary epitope on the assembled GnGc hexamer, with an extensive binding interface that cross-links two adjacent Gn monomers through 13 hydrogen bonds and 4 salt bridges [37]. The requirement for simultaneous mutations in two adjacent Gn protomers to disrupt antibody binding, combined with the functional constraint of maintaining the pre-fusion hexamer assembly, imposes a significantly higher genetic barrier to escape for RVFV-140 compared to antibodies targeting monomeric Gn epitopes. Similarly, the Gc-targeting nanobody NA137 binds to residues within and adjacent to the Gc fusion loop [42], a region that is structurally and functionally constrained, as it is essential for mediating pH-dependent membrane fusion, and is therefore highly conserved across RVFV strains.

The recent identification of LRP1 as the critical host entry receptor for RVFV has also provided a new, rational framework for epitope selection in antibody development [19]. The Gn-LRP1 interaction interface represents a functionally constrained “Achilles’ heel” of the virus, as mutations within this interface that disrupt antibody binding would likely also impair receptor binding and viral infectivity. As such, antibodies that directly target the LRP1 binding interface on Gn may achieve potent neutralization while exhibiting a high barrier to viral escape. However, to date, few reported RVFV NAbs have been shown to directly compete with LRP1 for Gn binding, and further structural and functional studies are needed to validate this approach and identify antibodies targeting this critical interface.

Conversely, it is worth noting that clinically circulating RVFV isolates display a remarkably high degree of sequence conservation, likely attributable to the relatively recent (100+ years) introduction of the virus into domestic ruminant populations [43]. Most reported escape mutations occur only in highly laboratory-passaged isolates (e.g., Ken56/IB8) and lack clinical relevance [44]. Furthermore, RVFV transmission depends primarily on enzootic cycles involving livestock and mosquito vectors, with humans playing no significant role in viral amplification [4]; consequently, even if an escape mutant were to arise in a treated patient, it would have minimal epidemiological consequences. Together with the potent in vivo protection observed in animal models, these factors suggest that the barrier to clinical deployment of mAb therapeutics against RVFV may be lower than for more variable viruses. Nonetheless, sustained surveillance for natural variants that could compromise antibody binding remains prudent, and the antibody engineering and combination strategies discussed below provide a practical framework to preempt such escape.

### 4.2. Rational Combination and Bispecific Antibody Strategies to Overcome Escape

Given the inherent propensity of RNA viruses to escape single-antibody selective pressure, combination antibody strategies—analogous to the combination antiretroviral therapy used for the treatment of HIV—are likely to be essential for the development of durable, broadly effective prophylactic and therapeutic interventions against RVFV. The synergistic neutralization and protective effect observed with the combination of Gn3 and Gn32 [34] provides early preclinical proof-of-concept that combining antibodies targeting distinct, non-overlapping epitopes on the viral glycoproteins can enhance neutralizing potency beyond additive effects. More importantly, such antibody combinations significantly raise the genetic barrier to viral escape, as the virus would need to acquire simultaneous, fitness-compatible mutations in multiple distinct epitopes to evade neutralization, which is statistically highly improbable.

Bispecific antibodies represent an elegant and clinically translatable approach to achieve the benefits of combination therapy within a single molecular entity, with simplified manufacturing, regulatory, and clinical development pathways compared to polyclonal antibody cocktails. The recently developed bispecific antibody E2-NA137, which simultaneously targets the Gn and Gc glycoproteins, provides a compelling proof-of-concept for this approach [42]. E2-NA137 exhibited a 270-fold enhancement in neutralizing potency compared to the parental NA137 nanobody, and provided significantly superior post-exposure therapeutic protection in the A129 mouse model (60% vs. 20% survival) [42]. This dual-targeting design not only enhances neutralizing efficacy through synergistic mechanisms, but also dramatically reduces the risk of viral escape, as the virus would need to mutate both the Gn and Gc epitopes simultaneously to evade neutralization.

Moving forward, future efforts should focus on the rational design of antibody cocktails or bispecific/multispecific molecules that combine antibodies with complementary mechanisms of action, including:

Antibodies targeting the receptor-binding interface on Gn to block viral attachment to host cells;

Antibodies targeting the fusion machinery (including the Gc fusion loop or quaternary Gn-Gc epitopes) to inhibit pH-dependent membrane fusion;

Non-neutralizing antibodies that enhance the potency of neutralizing antibodies through conformational modulation of the Gn-Gc complex (e.g., Gn32-like antibodies), or that mediate Fc-dependent effector functions such as antibody-dependent cellular cytotoxicity (ADCC) or antibody-dependent cellular phagocytosis (ADCP), which can contribute to viral clearance in vivo.

Notably, the majority of preclinical studies to date have focused exclusively on the neutralizing activity of RVFV antibodies, with minimal evaluation of Fc-mediated effector functions. Emerging evidence from other viral systems, including SARS-CoV-2 and Ebola virus, has demonstrated that Fc-mediated effector functions can significantly contribute to the in vivo protective efficacy of therapeutic antibodies. As such, future studies should systematically evaluate the contribution of Fcγ receptor engagement and effector functions to the protective efficacy of RVFV NAbs, and optimize the Fc region of lead candidates to enhance these functions, where appropriate.

### 4.3. Additional Translational Challenges

Beyond the challenge of viral immune escape, several additional hurdles must be overcome to translate RVFV NAbs from preclinical studies into clinical use. First, the stability and manufacturability of antibody candidates are critical considerations, particularly given that RVFV is endemic primarily in resource-limited regions of Africa and the Arabian Peninsula, where cold-chain infrastructure is often limited. Conventional IgG antibodies require strict cold-chain storage and transport to maintain stability and biological activity, which presents significant logistical and economic barriers to deployment in endemic settings. In this regard, single-domain nanobodies such as NA137 offer significant advantages, as they exhibit exceptional thermal and chemical stability, and can be manufactured at low cost in microbial expression systems, without the need for cold-chain storage [42]. Future efforts should prioritize the development of thermostable antibody formats, including nanobodies, bispecific nanobodies, and Fc-fusion nanobodies, which are better suited for deployment in resource-limited endemic regions.

Second, the observation that RVFV-268 fails to completely neutralize the virus in certain cell lines, including HEK-293 and HepG2 [19], highlights that tissue-specific and cell-type-pecific factors can influence the efficacy of neutralizing antibodies in vivo. RVFV exhibits broad tropism, infecting multiple tissues including the liver, brain, spleen, and placenta, and antibodies must be able to efficiently penetrate these tissues and neutralize the virus in diverse cell types to provide comprehensive protection. This underscores the importance of evaluating lead antibody candidates in a panel of physiologically relevant cell types, including primary human hepatocytes, neuronal cells, and placental cells, as well as in animal models that faithfully recapitulate the full spectrum of human RVF disease, including hepatic failure, encephalitis, and vertical transmission.

Third, the clinical development of RVFV therapeutics is hindered by the sporadic and unpredictable nature of RVF outbreaks, which makes large-scale, randomized controlled clinical trials challenging to design and execute. To address this barrier, future clinical development programs should leverage the U.S. Food and Drug Administration (FDA) Animal Rule, which allows for the approval of medical countermeasures for emerging infectious diseases based on well-controlled animal efficacy studies, when human efficacy trials are not feasible or ethical. This will require the establishment of well-characterized animal models that are predictive of human disease, and the standardization of preclinical study endpoints to support regulatory approval.

Finally, the potential for antibody-dependent enhancement (ADE) of infection, a phenomenon in which sub-neutralizing concentrations of antibodies facilitate viral entry into Fc receptor-expressing cells, leading to enhanced disease severity, must be rigorously evaluated for all lead antibody candidates. Although classical ADE has not been conclusively demonstrated for RVFV, early studies have noted that passive transfer of certain immune sera could exacerbate disease in experimentally infected mice, raising the possibility of antibody-mediated enhancement phenomena. Notably, our group recently observed direct evidence of an ADE-like effect in another bunyavirus. During the development of a rVSV-HTNV-GP-based surrogate neutralization assay for Hantaan orthohantavirus (HTNV), one convalescent HFRS patient serum (NO. 11) consistently enhanced infection at low dilutions (1:10 to 1:80) rather than neutralizing the virus [45]. Together with existing literature reporting ADE in hantavirus-infected macrophage cell lines [46], this finding further underscores the potential susceptibility of bunyaviruses to antibody-mediated enhancement. As such, comprehensive in vitro and in vivo studies are required to exclude the risk of ADE for lead RVFV antibody candidates prior to clinical evaluation, as documented for other viruses, most notably dengue virus and, more recently, SARS-CoV-2 [47,48,49].

## 5. Conclusions

In summary, the field of RVFV neutralizing antibody research has advanced at a remarkable pace over the past decade, with a large panel of potent neutralizing antibodies identified from diverse sources, including immunized rodents, rabbits, alpacas, non-human primates, and convalescent human patients. Structural and functional studies have elucidated two primary mechanisms of action for these antibodies: (1) blockade of viral attachment to host cells, either by direct competition with the LRP1 receptor binding interface or by steric masking of the receptor binding site; and (2) inhibition of pH-dependent membrane fusion, by stabilizing the pre-fusion conformation of the Gn-Gc complex and preventing the conformational rearrangement required for Gc fusion loop exposure. The recent discovery of bispecific antibodies with dual-targeting activity, and antibodies that act through a dual mechanism of blocking both attachment and fusion, has further expanded the repertoire of promising candidates, with enhanced potency and a reduced risk of viral escape.

The epitope landscape on the Gn glycoprotein is now well characterized, with at least four major neutralizing antigenic sites defined—domains A, B, C, and the Gn-Gc quaternary interface—each exemplified by structurally characterized antibodies including RVFV-268 and R15 (domain A), RV-Gn1 (domain B), R17 and 1332F11 (domain C), and RVFV-140 (quaternary epitope). In contrast, the Gc glycoprotein has yielded fewer independent neutralizing antibodies; however, the recent successful targeting of the Gc fusion loop by NA137 and the bispecific E2-NA137 demonstrates that Gc remains a viable therapeutic target, especially when used in combination with Gn-directed agents.

These advances have not only provided critical insights into the humoral immune response against RVFV and the structural basis of viral neutralization, but have also yielded a panel of high-quality lead candidates for the development of prophylactic and therapeutic interventions against RVF. Fully human antibodies such as RVFV-268 and RVFV-140 have demonstrated potent protective efficacy in multiple animal models, including against aerosol challenge and congenital infection, supporting their further development for clinical use.

However, significant challenges remain to be addressed, including the risk of viral immune escape, the need for thermostable antibody formats suitable for deployment in resource-limited settings, and the need for rigorous preclinical evaluation to support regulatory approval via the Animal Rule. Moving forward, a multi-pronged approach combining structure-guided antibody engineering, rational design of antibody combinations and bispecific molecules, systematic evaluation of Fc-mediated effector functions, and rigorous testing in clinically relevant animal models will be critical to overcome these challenges, and translate these promising preclinical candidates into effective, licensed interventions to address the unmet medical need for RVF prevention and treatment.

## Figures and Tables

**Table 1:** Target specificity, neutralizing potency, and preclinical research progress of neutralizing antibodies against Rift Valley fever virus (RVFV).

Group (Antibody Source)	Antibody Name	Target Region on Viral Glycoproteins	Neutralization Potency (IC_50_, ng/mL)	Key Preclinical Research Findings	Reference
Group A Rabbit—derived mAbs ^1^	RV—Gn1	Gn Domain B	2100–3000	100% survival in BALB/c mice with pre—exposure prophylaxis; neutralizes via blocking Gc fusion loop exposure; conserved binding mode with anti—SFTSV human mAbs	[35]
	RV—Gn2	Gn Domain B	2100–3000	In vitro neutralizing activity validated; clonally related to RV—Gn1 with overlapping epitope	[35]
	RV—Gn3	Gn Domain B	2100–3000	In vitro neutralizing activity validated; clonally related to RV—Gn1 with overlapping epitope	[35]
Group B Murine—derived hybridoma mAbs ^2^	Gn3	Undetermined Gn epitope	33,000	Moderate protective efficacy in BALB/c mice (58.3% survival with monotherapy); 83.3% (pre—exposure) and 100% (post—exposure) survival when combined with non—neutralizing mAb Gn32; synergistic effect via Gn conformational modulation	[21]
	Gn32	Gn N—terminal distal region	ND	No intrinsic neutralizing activity; enhances Gn3 potency via inducing Gn conformational change	[21]
Group C Fully human mAbs (convalescent/MP—12 vaccinated individuals) ^3^	RVFV—268	Gn Domain A	0.2	Significant therapeutic benefit when administered 2 days post—infection in mice; 72% survival in rat aerosol challenge model; effectively prevents vertical transmission in utero; susceptible to Gn T173L/E175G/K294E—D230N escape mutations	[17,19,36]
	RVFV—140	Gn—Gc hexamer quaternary epitope	13	100% neutralization across multiple human cell lines; 83% survival in rat aerosol challenge model (highest among tested mAbs); neutralizes via stabilizing pre—fusion Gn—Gc complex to block membrane fusion; high genetic barrier to viral escape	[7,19,37]
	RVFV—144	Gn—Gc complex	3330	In vitro neutralizing activity validated; in vivo protective efficacy comparable to RVFV—268 in mouse models	[17]
Group D Non—human primate (NHP)—derived mAbs ^4^	A38	Gn Domain I & III	0.76	100% survival in A129 mice with both prophylactic (high/low dose) and therapeutic (high/low dose) administration; neutralizes via dual mechanism: blocks viral attachment and inhibits membrane fusion	[38]
	A13	Gn Domain I, II & III	208.90	100% survival in A129 mice with prophylactic high—dose administration; 100% survival with therapeutic high—dose administration; neutralizes via inhibiting membrane fusion	[38]
	E35—1	Gn Domain I	1.44	In vitro neutralizing activity validated; no in vivo efficacy data reported	[38]
	E35—4	Gn Domain I	1.54	In vitro neutralizing activity validated; no in vivo efficacy data reported	[38]
	D20	Gn Domain I	1.48	In vitro neutralizing activity validated; no in vivo efficacy data reported	[38]
	E18	Gn Domain I	1.83	In vitro neutralizing activity validated; no in vivo efficacy data reported	[38]
	A37	Gn Domain I	1.95	In vitro neutralizing activity validated; no in vivo efficacy data reported	[38]
	E32	Gn Domain I	2.72	In vitro neutralizing activity validated; no in vivo efficacy data reported	[38]
	E33	Gn Domain I	3.52	In vitro neutralizing activity validated; no in vivo efficacy data reported	[38]
	A36	Gn Domain I	4.92	In vitro neutralizing activity validated; no in vivo efficacy data reported	[38]
	E38	Gn Domain I	9.26	In vitro neutralizing activity validated; no in vivo efficacy data reported	[38]
	F15	Gn Domain III	1.94	In vitro neutralizing activity validated; no in vivo efficacy data reported	[38]
	C16	Gn Domain III	9.53	In vitro neutralizing activity validated; no in vivo efficacy data reported	[38]
	F16	Gn Domain III	103.40	In vitro neutralizing activity validated; no in vivo efficacy data reported	[38]
	E44	Gn Domain III	56.37	In vitro neutralizing activity validated; no in vivo efficacy data reported	[38]
	A20	Gn Domain III	285.90	In vitro neutralizing activity validated; no in vivo efficacy data reported	[38]
	E17	Gn Domain III	84.79	In vitro neutralizing activity validated; no in vivo efficacy data reported	[38]
	E5	Gn Domain III	618.70	In vitro neutralizing activity validated; no in vivo efficacy data reported	[38]
	F11	Gn Domain III	12.40	In vitro neutralizing activity validated; no in vivo efficacy data reported	[38]
	E3	Gn Domain III	83.40	In vitro neutralizing activity validated; no in vivo efficacy data reported	[38]
Group E Fully human mAbs (imported convalescent case) ^5^	R16	Gn Domain C	0.29	100% survival in BALB/c mice with both pre—exposure and post—exposure administration	[39]
	R15	Gn Domain A & B	0.53	100% survival in BALB/c mice with both pre—exposure and post—exposure administration; neutralizes via blocking Gn—host receptor interaction	[39]
	R12	Gn Domain A & B	1.85	100% survival in BALB/c mice with both pre—exposure and post—exposure administration; neutralizes via blocking Gn—host receptor interaction	[39]
	R17	Gn Domain B & C	2.53	100% survival in BALB/c mice with both pre—exposure and post—exposure administration; neutralizes via inhibiting membrane fusion	[39]
	R4	Gn Domain A	35.1	100% survival in BALB/c mice with both pre—exposure and post—exposure administration	[39]
	R13	Gn Domain A & B	48.4	100% survival in BALB/c mice with both pre—exposure and post—exposure administration	[39]
	R19	Gn Domain B	48.3	100% survival in BALB/c mice with both pre—exposure and post—exposure administration	[39]
	R22	Gn Domain A & B	76.3	100% survival with pre—exposure administration; 75% survival with post—exposure administration in BALB/c mice	[39]
	R5	Gc	1370	Only 20% survival in BALB/c mice with both pre—exposure and post—exposure administration; contributes to viral clearance at high concentrations	[39]
Group F NHP—derived mAbs ^6^	1331E4	Gn Domain C	338.8	High Gn—binding affinity validated; in vitro neutralizing activity confirmed; putative mechanism via blocking Gn—Gc conformational rearrangement; in vivo efficacy remains to be determined	[40]
	1332F11	Gn Domain C	167.8	In vitro neutralizing activity confirmed; putative mechanism via blocking Gn—Gc conformational rearrangement; in vivo efficacy remains to be determined	[40]
Group G Murine—derived mAbs (single—cell sequencing) ^7^	Gn1—Gn14	Gn	ND	High—affinity Gn binding validated (Kd as low as 0.65 nM); in vitro neutralizing activity partially characterized	[41]
	Gc1—Gc9	Gc	ND	High—affinity Gc binding validated (Kd as low as 2.1 nM); in vitro neutralizing activity partially characterized	[41]
Group H Alpaca—derived nanobody & bispecific antibody	NA137	Gc fusion loop	2701	80% survival in A129 mice with pre—exposure prophylaxis; 20% survival with post—exposure administration; targets conserved Gc fusion loop	[42]
	E2—NA137	Gn + Gc (bispecific)	10	80% survival in A129 mice with pre—exposure prophylaxis; 60% survival with post—exposure administration; 270—fold enhanced potency vs. parental NA137; dual—targeting design reduces viral escape risk	[42]

^1^ Group A: Neutralizing activity was determined by plaque reduction neutralization test (PRNT). In vivo efficacy was evaluated in female BALB/c mice challenged with 20 PFU of RVFV strain ZH501, with antibody administered intravenously 8 h prior to challenge. Target epitopes were determined by co—crystal structure analysis. ^2^ Group B: Neutralizing activity was determined by serum neutralization assay. In vivo efficacy was evaluated in BALB/c mice challenged with RVFV strain 35/74, with antibody administered via tail vein injection 30 min pre— or post—challenge. Target epitopes were determined by epitope mapping. ^3^ Group C: Neutralizing activity was determined by focus—forming assay (FFA) against wild—type RVFV strain ZH501. In vivo efficacy was evaluated in C57BL/6 and BALB/c mice challenged with 300 PFU of ZH501, as well as Lewis rat aerosol challenge and congenital infection models. Target epitopes were determined by X—ray crystallography and cryo—electron microscopy. ^4^ Group D: Neutralizing activity was determined by microneutralization assay against RVFV strain rMP—12—eGFP. In vivo efficacy was evaluated in interferon—α/β receptor—deficient A129 mice challenged with 2 × 10^4^ TCID_50_ of RVFV rMP—12, with antibody administered intraperitoneally 24 h pre— or post—challenge. ^5^ Group E: Neutralizing activity was determined by flow cytometry—based neutralization assay in Vero cells. In vivo efficacy was evaluated in BALB/c mice challenged with 1 × 10^3^ PFU of RVFV, with antibody administered intraperitoneally 24 h pre— or post—challenge. Target epitopes were determined by co—crystal structure and mutational analysis. ^6^ Group F: Neutralizing activity was determined using a Renilla luciferase—expressing RVFV MP—12 strain. ^7^ Abbreviations: IC_50_, half maximal inhibitory concentration; PFU, plaque—forming unit; TCID_50_, 50% tissue culture infectious dose; PRNT, plaque reduction neutralization test; FFA, focus—forming assay; ND, not determined; mAb, monoclonal antibody; SFTSV, severe fever with thrombocytopenia syndrome virus.

## Data Availability

No new data were created or analyzed in this study.
